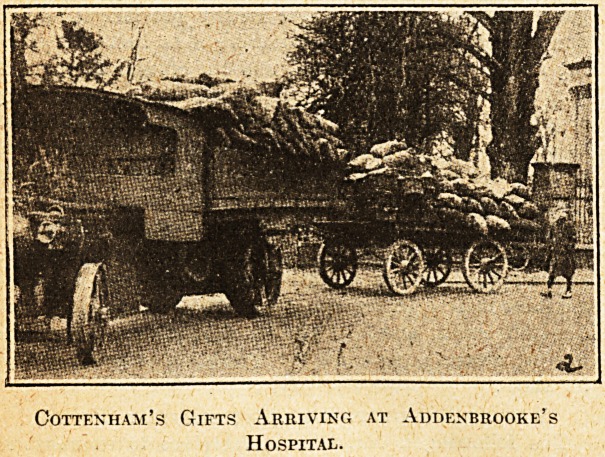# The Organisation of Gifts in Kind

**Published:** 1918-01-12

**Authors:** 


### INCOME BY HUNDREDWEIGHT.

### The Organisation of Gifts in Kind.

It is not sufficiently realised by hospital authorities how much their institution can be benefited by gifts in kind. There are certain sections of the community interested in our hospitals which give their support in the form most convenient to them—that is to say, in gifts in kind. It is significant that such a source of assistance has remained but little developed for so long. An instance of what can be done in this direction may be cited. Addenbrooke's Hospital, Cambridge, has received this year from the villages of Isleham, Burwell, Cottenham, and Willingham gifts of

Cottenham's Gifts Arriving at Addenbrooke's Hospital.

(From the *Cambridge Chronicle* and *University Journal*.)

fruit and vegetables of every description, as well as a certain amount of groceries and provisions, including jam, etc., weighing about 40 tons, together with about £22 in cash, equal in total value to a gift of £350 at least.

From these village collections of gifts in kind and from Harvest Thanksgiving offerings Addenbrooke's Hospital has this year received about fifty tons of potatoes. Hospitals generally would do well to develop this productive source of support if they have not already done so.

The method adopted at Cambridge has been for the secretary superintendent, when attending various Hospital Sunday parades during the summer and autumn, to address open-air meetings with a view to creating greater interest in the country districts. It was recognised that there were certain villages served by the hospital where for some reason or local tradition there was little hope of obtaining much more support in money. Nevertheless, in such villages, where for the most part nearly everyone is occupied in fruit growing and market gardening, there was abundant opportunity of securing additional assistance in fruit and vegetables. Mr. Coles seized the opportunity opened up, and made an appeal at Cottenham, among other villages, for gifts in kind. At the time his appeal appeared to have fallen on deaf ears. But imagine the pleasant surprise when, about six weeks after his appeal was made, two messengers called at the hospital and informed him that they had brought the fruit, potatoes, and other vegetables which they had collected in response to his appeal. Much greater was the surprise when he observed a steam traction engine with two large lorries laden with six tons of potatoes, one ton of carrots, one ton of apples, ½ ton of beet and turnips, besides onions, celery, and greens. In addition to these the sum of £12 15s. 6d. was contributed by those residents in the village unable to give in kind, besides the sum of £7 8s. raised by a Flag Day. The total result of this collection of gifts in kind for the first year amounts to £98 16s.

The Cottenham Friendly Societies' Parade Committee, consisting of Messrs W. Collins (chairman), A. W. Young (secretary), H. Furbank (treasurer), and their colleagues, in response to the appeal by Mr. Coles, arranged a house-to-house canvass in Cottenham for promises of gifts of fruit and vegetables to be sent to a collecting depôt provided by Mr. C. Bird, with the excellent result above mentioned, and Messrs. C. Lane Sons lent their steam traction engine and lorries to convey the gifts to Addenbrooke's. It is hoped that this brief account of how it was done may prove an incentive to other hospitals.

## Figures and Tables

**Figure f1:**